# Effects of inorganic nitrate on ischaemia-reperfusion injury after coronary artery bypass surgery: a randomised controlled trial

**DOI:** 10.1016/j.bja.2021.06.046

**Published:** 2021-08-14

**Authors:** Karin E. Eriksson, Fredrik Eidhagen, Jan Liska, Anders Franco-Cereceda, Jon O. Lundberg, Eddie Weitzberg

**Affiliations:** 1 Department of Physiology and Pharmacology, Karolinska Institutet, Stockholm, Sweden; 2 Department of Perioperative Medicine and Intensive Care, Karolinska University Hospital, Stockholm, Sweden; 3 Stockholm Center for Spine Surgery (RKC), Stockholm, Sweden; 4 Department of Molecular Medicine and Surgery, Karolinska Institutet, Stockholm, Sweden; 5 Department of Cardiothoracic Surgery, Karolinska University Hospital, Stockholm, Sweden

**Keywords:** bleeding, cardiac surgery, ischaemia-reperfusion injury, nitric oxide, nitrate, nitrite, perioperative

## Abstract

**Background:**

Nitric oxide (NO) is an important signalling molecule in the cardiovascular system with protective properties in ischaemia–reperfusion injury. Inorganic nitrate, an oxidation product of endogenous NO production and a constituent in our diet, can be recycled back to bioactive NO. We investigated if preoperative administration of inorganic nitrate could reduce troponin T release and other plasma markers of injury to the heart, liver, kidney, and brain in patients undergoing cardiac surgery.

**Methods:**

This single-centre, randomised, double-blind, placebo-controlled trial included 82 patients undergoing coronary artery bypass surgery with cardiopulmonary bypass. Oral sodium nitrate (700 mg×2) or placebo (NaCl) were administered before surgery. Biomarkers of ischaemia–reperfusion injury and plasma nitrate and nitrite were collected before and up to 72 h after surgery. Troponin T release was our predefined primary endpoint and biomarkers of renal, liver, and brain injury were secondary endpoints.

**Results:**

Plasma concentrations of nitrate and nitrite were elevated in nitrate-treated patients compared with placebo. The 72-h release of troponin T did not differ between groups. Other plasma biomarkers of organ injury were also similar between groups. Blood loss was not a predefined outcome parameter, but perioperative bleeding was 18% less in nitrate-treated patients compared with controls.

**Conclusion:**

Preoperative administration of inorganic nitrate did not influence troponin T release or other plasma biomarkers of organ injury in cardiac surgery.

**Clinical trial registration:**

NCT01348971.

**Editor's key points**

• Nitrate or nitrite have been shown to be protective against ischaemia–reperfusion injury.
• This randomised trial investigated whether oral nitrate before cardiac surgery could protect against ischaemia–reperfusion injury in cardiac surgery.
• Biomarkers for ischaemia–reperfusion injury, including troponin T, did not differ between pretreated and control groups.
• This study does not support the preoperative use of dietary nitrate to reduce organ injury related to ischaemia–reperfusion injury in cardiac surgery.

A variety of pharmacological and mechanical strategies aimed at reducing myocardial ischaemia–reperfusion (IR) injury after coronary artery bypass grafting (CABG) have been proposed, but with limited success.^1^, ^2^, ^3^ Myocardial injury is monitored by release of biomarkers such as troponin T and creatine phosphokinase-myocardial band (CK-MB) and elevated concentrations of circulating troponin have been associated with cardiac events and increased mortality.^4^^,^^5^

Nitric oxide (NO) is a pluripotent signalling molecule widely produced in the cardiovascular system where it upholds homeostasis by its vasodilatory, anti-aggregatory, and anti-adhesive actions.^6^ Generation of NO is achieved by specific NO synthases (NOSs) but NO bioavailability is diminished during aging and in many cardiovascular disorders.^7^ In various experimental models of IR injury, NO has been shown to have protective properties but clinical studies are scarce.^8^ The organic nitrates such as glyceryl trinitrate (nitroglycerin, GTN), used in cardiovascular medicine, act by generating NO but are limited by the development of tolerance and endothelial dysfunction.^9^^,^^10^ Perioperative nitroglycerin in CABG has been evaluated in several studies, but a recent meta-analysis concludes that the protective effects are very limited.^11^ Nevertheless, new studies evaluating nitroglycerin in CABG are ongoing.^12^

In addition to NOS-dependent NO generation, NO and other reactive nitrogen oxides can be produced by reduction of the inorganic anions nitrate and nitrite.^13^ The bioavailability of nitrate after oral intake is almost 100%. Interestingly, 25% of a circulating nitrate is actively taken up in the salivary glands and excreted in saliva, after which oral bacteria converts nitrate to nitrite. Systemically there are several pathways for further reduction of nitrite to bioactive NO. This nitrate-nitrite-NO pathway is enhanced in situations of hypoxia and low pH, in contrast to the NOSs that perform poorly under such circumstances. A significant source of nitrate is our diet, where green leafy vegetables are naturally rich in this anion.^14^

There are now several animal studies showing beneficial effects of nitrate and nitrite in models of cardiac IR injury.^15^, ^16^, ^17^ Nitrite has been suggested as an ischemic preconditioning mimetic^18^ and also to be contributing to the effects of remote preconditioning.^19^ Because of the much longer half-life of nitrate (5–6 h) compared with nitrite (20–30 min), nitrate may be considered as a pro-drug to achieve a more prolonged elevation of nitrite in blood and tissues. Mechanistically it has been shown that the cytoprotective effects are likely attributable to S-nitrosation of complex I in the mitochondrial respiratory chain, leading to less formation of reactive oxygen species (ROS) and reduced cytochrome C release.^20^^,^^21^ Other possible beneficial effects of nitrate and nitrite in the pathophysiology of IR injury are vasodilation,^22^ inhibition of platelet aggregation,^23^ anti-adhesion of white blood cells,^24^ improved endothelial function,^25^ and inhibition of nicotinamide adenine dinucleotide phosphate (NADPH) oxidase (NOX) generation of ROS.^26^

Two human trials have explored intravenous (i.v.) or intracoronary administration of nitrite in patients with acute myocardial infarction treated with percutaneous coronary intervention. While i.v. administration lacked protective effects,^27^ intracoronary nitrite positively affected arrhythmias in the acute setting and major adverse cardiac events up to one year later.^28^ In a more recent study, i.v. administration of nitrite given by paramedics during resuscitation for out-of-hospital cardiac arrest did not improve survival to hospital admission.^29^

In this study, we hypothesised that preoperative inorganic nitrate could reduce troponin T release and other biomarkers of cardiac, renal, hepatic, and cerebral injury in patients undergoing cardiac surgery.

## Methods

### Trial design

The Coronary Artery Bypass and Nitrate Oral Supplementation (CABANOS) trial is a single centre, randomised, placebo-controlled, and double-blinded study. The trial was approved by the Swedish Ethical Review Authority in Stockholm. Patients planned for CABG with cardiopulmonary bypass (CPB) who gave their written informed consent were included in the study.

### Study population

All patients with coronary artery disease planned for CABG with CPB were screened for eligibility. Patients between 18 and 80 years of age were eligible for enrolment. Exclusion criteria were pregnancy, re-do surgery, ongoing angina or troponin release above 45 ng L^−1^ <48 h before surgery, intended heart valve or additional surgery, medication with organic nitrates/nitrites, glibenclamide, steroids <24 h before surgery, or significant renal, pulmonary, or hepatic disease. In general, patients suffered from other cardiovascular co-morbidities such as hypertension, diabetes mellitus, hyperlipidaemia, and in some cases heart failure (Table 1). Almost all patients were treated with acetylsalicylic acid which was kept until the day of surgery. Seven patients in the placebo group and nitrate treatment group, respectively, were treated with clopidogrel, but this was paused at least 5 days before surgery.

**Table 1:** Subject characteristics at baseline, medication, perioperative factors, and scoring. Data are shown as median (inter-quartile range) and numbers (%). The groups are compared with the Mann–Whitney *U*-test and Fisher's exact test. ACE, angiotensin converting enzyme; BMI, body mass index; CPB, cardiopulmonary bypass, EF, ejection fraction.

Characteristics	Placebo (*n*=42)	Nitrate (*n*=40)	*P*
Age, yr	67 (60–71)	66 (60–69)	0.44
Male sex	40 (95.2)	35 (87.5)	0.26
BMI, kg m^-2^	27.7 (25.5–29.6)	26.8 (26.0–30.0)	0.94
Diabetes mellitus	11 (26.2)	8 (20.0)	0.60
Euroscore	1.8 (1.1–3.3)	1.6 (1.0–2.3)	0.19
EF<45%	9 (21.4)	6 (15.0)	0.57
Smoking	2	1	0.61
ACE inhibition	23 (54.8)	20 (50.0)	0.83
Angiotensin receptor blocker	5 (11.9)	11 (27.5)	0.10
Calcium blocker	9 (21.4)	10 (25.0)	0.80
Beta blocker	37 (88.1)	39 (97.5)	0.20
Acetylsalicylic acid	41 (97.6)	40 (100)	1.0
Clopidogrel	7 (16.7)	7 (17.5)	1.0
Statins	40 (95.2)	39 (97.5)	1.0
Metformin	5 (11.9)	3 (7.5)	0.71
Main stem stenosis	12 (28.5)	17 (42.5)	0.25
Instable angina	7 (16.7)	10 (25.0)	0.42
Number of grafts	3 (2–4)	3 (2–3)	0.23
Occlusion time (min)	40 (29–66)	42 (29–51)	0.37
CPB time (min)	63 (47–91)	66 (49–80)	0.89
Higgins score	0 (0–4)	0 (0–3)	0.42
Reoperation	2 (4.8)	0 (0)	0.49
Ventilator time (min)	136 (104–213)	120 (70–170)	0.12

### Randomisation and intervention

Patients who met enrolment criteria and provided written informed consent were randomly assigned, in blocks of four, to intervention or placebo. Subjects in the placebo group were treated with two doses of orally administered sodium chloride (NaCl, 700 mg), the first one in the evening before surgery and the second dose 3 h before start of anaesthesia. The participants in the nitrate group were at the same time points given peroral sodium nitrate (NaNO_3_, 700 mg). Subjects, attending anaesthesiologists, personnel who collected data, and personnel who assessed outcomes were unaware of the trial-group assignments.

### Procedures

Enrolled subjects were served a standardised, low-nitrate meal (15 [5] μmol nitrate per portion) the evening before surgery. All subjects were anaesthetised and monitored in a standardised manner. ECG, pulse oximetry, and invasive BP measurement were used in all patients and started before induction. Anaesthesia was induced with midazolam (approximately 40 μg kg^−1^), fentanyl (approximately 7 μg kg^−1^), and propofol (1.5 mg kg^−1^ BW) and the patients were intubated orally after relaxation with atracurium (0.5 mg kg^−1^ BW). All patients received a central venous catheter via the right jugular vein and were examined with transoesophageal echocardiogram. Anaesthesia was maintained with sevoflurane (minimum alveolar concentration 0.8–1.2) until start of extracorporeal circulation, when there was a shift to infusion of propofol (4–6 mg kg^−1^ h^−1^). After weaning of CPB, volatile anaesthetics were again used. All subjects were treated with the antifibrinolytic drug tranexamic acid and prophylactic cloxacillin perioperatively. Subjects with blood glucose >10 mmol L^−1^ were treated with infusion of short-acting insulin to keep blood glucose between 5 and 10 mmol L^−1^.

Coronary artery bypass surgery was performed through median sternotomy. Standard dissection of a pedicled left internal thoracic artery was used as arterial graft and standard dissection of the saphenous vein performed for the use of vein grafts. CPB was initiated after heparinisation by arterial cannulation in the ascending aorta and venous cannulation in the right atrium. Systemic temperature was maintained between 36°C and 37°C. The aorta was cross-clamped, and antegrade cold blood cardioplegia administered every 15 min. Central venous anastomosis was performed with a partial clamp of the ascending aorta. The effect of heparin was reversed by protamine. Perioperative bleeding was measured.

Postoperatively, the sedated patients were transferred to the cardiothoracic ICU (CICU) for continued observation, weaned from mechanical ventilation, and extubated. Most subjects needed overnight monitoring in the CICU before transferral to the ward. The collected amount from the chest drains was summarised as the postoperative bleeding. Some subjects suffered from complications such as respiratory failure, atrial fibrillation, and infections (Supplementary Table S1). This minority of subjects got prolonged support in the CICU and treatment as needed. Physiological parameters and plasma samples were collected perioperatively and for up to 72 h after surgery. Plasma nitrate and nitrite were analysed from the samples obtained at start of anaesthesia and at the end of extracorporeal circulation.

### Biochemical analysis

Analysis of troponin T, creatine phosphokinase-myocardial band (CK-MB), NT-proB-type natriuretic peptide (pro-BNP), creatinine, cystatin C, alanine aminotransferase (ALT), aspartate aminotransferase (AST), bilirubin, high sensitivity C-reactive protein (hsCRP), neuron specific enolase (NSE), and S100B protein (S100B) was performed by the accredited chemical laboratory at Karolinska Hospital.

Nitrate concentrations in the meals served were determined by chemiluminescence after reductive cleavage and subsequent release of NO into the gas phase as described earlier.^30^ For measurements of nitrate and nitrite in plasma samples we used a previously described sensitive and selective high performance liquid chromatography (HPLC) system (ENO-20) and autosampler (840, EiCom, Kyoto, Japan), which uses reverse phase chromatography to separate nitrite from nitrate, and then nitrate is reduced to nitrite through a reaction with cadmium and reduced copper inside a reduction column.^31^

### Endpoints and outcome measures

The predefined primary endpoint was troponin T release over the perioperative 72-h period. Our secondary endpoints were release of troponin T within 24 h postoperatively and changes of the following measurements over the perioperative 72-h period: CK-MB, pro-BNP, creatinine, cystatin C, AST, ALT, ALP, bilirubin, and hsCRP C, NSE, and S100B. In addition, we observed and documented perioperative vital parameters, perioperative and postoperative bleeding (summarised as the total bleeding), and upcoming complications of any type.

### Statistical analysis

Normal distributed data such as circulatory parameters are presented as mean and standard deviation (sd) and otherwise as median with inter-quartile range.

A sample size of at least 40 subjects in each group was needed to have a power >90%, an sd of 250 ng L^−1^, significance at the two-sided 5% level, and on the basis of other intervention studies^3^ we expected a difference in plasma troponin T of 200 ng L^−1^ between the groups.

To compare the two groups, we used the non-parametric Mann–Whitney test. Contingency data were analysed with the Fisher's exact test. In cases with repeated measurements over time we used mixed model analysis for comparison of changes relative to baseline within and between groups. To test the association between nitrate and nitrite with troponin T we used the Pearson correlation coefficient. IBM SPSS Statistics version 27 (IBM, New York, United States) was used for data processing and statistical analysis, and for figures and some analysis we applied GraphPad Prism 8 (GraphPad Software, San Diego, United States).

## Results

### Trial population and baseline characteristics

**Fig 1:**
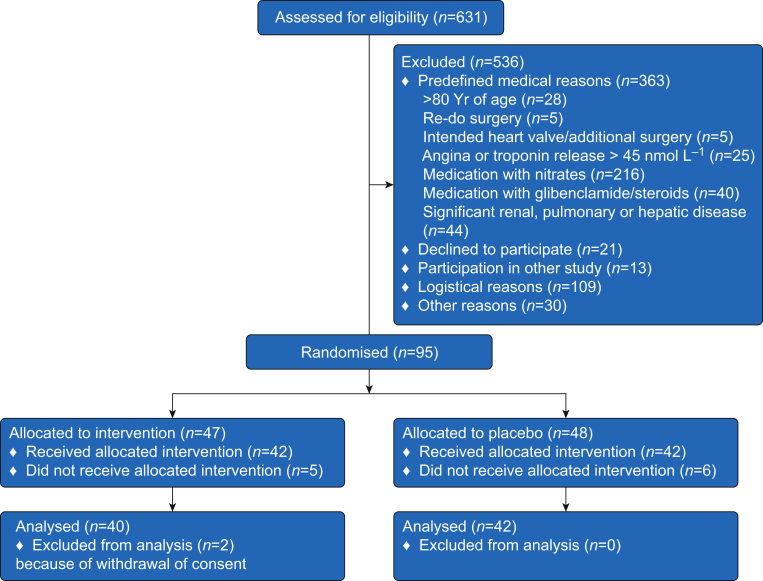
Enrolment and randomisation in the CABANOS trial. Patients were randomised in blocks of four to intervention with sodium nitrate or sodium chloride (placebo) before coronary artery bypass surgery.

We screened, in total, 631 patients for eligibility and 536 were excluded because of not meeting the inclusion criteria or lack of informed consent (Fig. 1). Ninety-five patients were randomised to invention or placebo after which 42 and 40 in the placebo group and nitrate group, respectively, completed the study protocol. As shown in Table 1, there were no differences between the two groups at baseline in terms of patient characteristics, medication, and subsequent perioperative parameters including occlusion time, CPB time, and Higgins score.

### Plasma nitrate and nitrite

**Fig 2:**
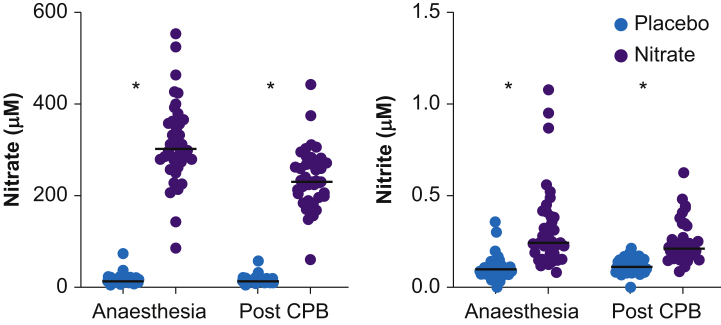
Plasma nitrate (a) and nitrite (b) in the placebo and nitrate groups after induction of anaesthesia and at the end of cardiopulmonary bypass (CPB). Individual values and line indicating median. ∗*P*<0.001.

To ensure that the nitrate-treated subjects had adhered to treatment we analysed plasma concentrations of nitrate and nitrite (Fig. 2). Plasma nitrate was higher in the nitrate-treated subjects with a peak median value of 302 (274–362) μM compared with 14 (11–20) μM in the placebo group. The corresponding nitrite values were 245 (161–389) nM and 98 (72–127) nM, respectively. In the nitrate-treated subjects both anions decreased during surgery but were still higher compared with placebo.

### Cardiac biomarkers

**Fig 3:**
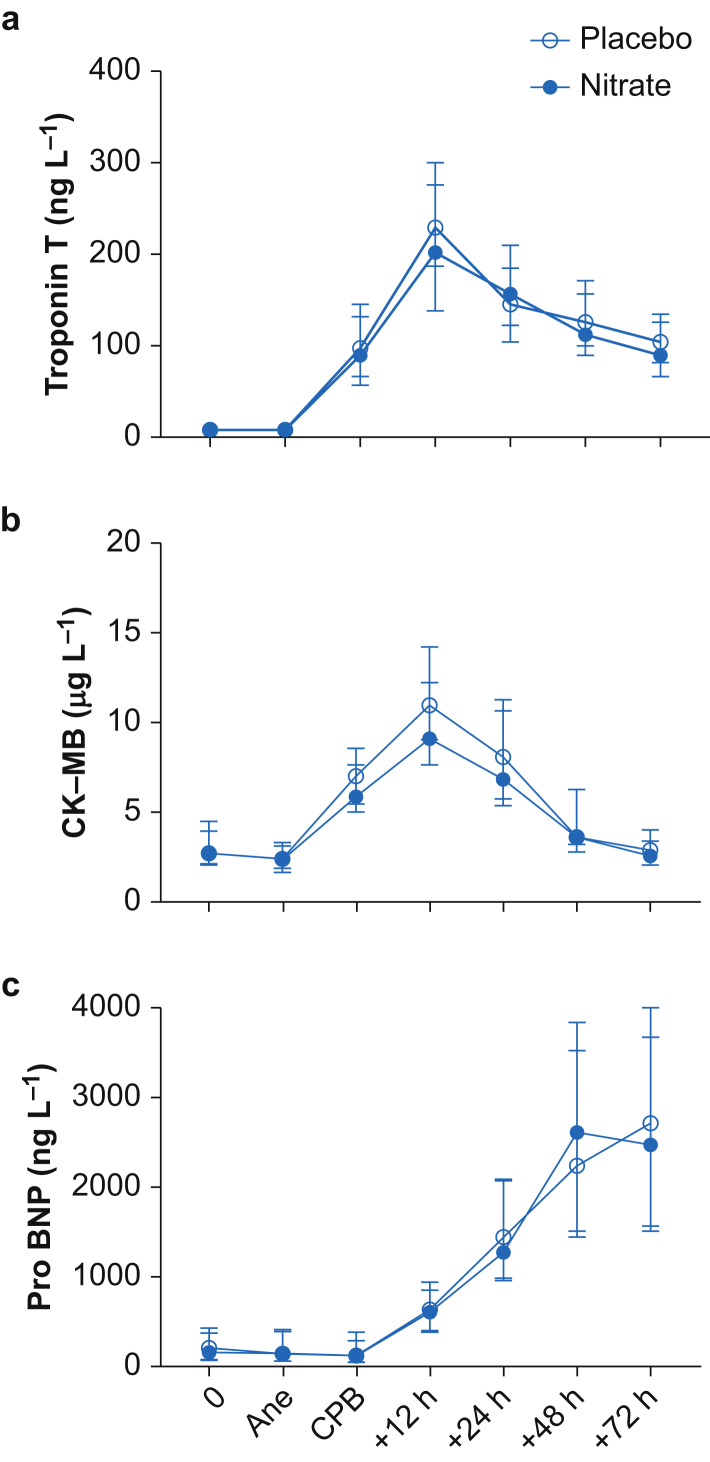
The cardiac biomarkers troponin T, creatine phosphokinase-myocardial band (CK-MB) and natriuretic peptide proB-type (pro-BNP) during the 72-h perioperative period. 0=baseline. Data are shown in median and inter-quartile range (IQR). Ane, start of anaesthesia; CPB, end of cardiopulmonary bypass.

Our main outcome parameter, troponin T, increased significantly in both groups during the 72-h postoperative period (Fig. 3). It peaked 12 h after CPB with a median of 203 (138–300) ng L^−1^ in the nitrate-treated group and 230 (177–305) ng L^−1^ in the placebo group (*P*=0.37). Mixed model analysis of the 72-hr period did not show any difference between the groups in troponin T release (*P*=0.19). There was no correlation between plasma nitrate or nitrite and total troponin T release (*r*=0.026, *P*=0.87 for nitrate and *r*=0.22, *P*=0.17 for nitrite). CK-MB and pro-BNP increased in a similar way in both groups, regardless of the intervention (Fig. 3).

### Renal, hepatic, brain and inflammatory biomarkers

**Fig 4:**
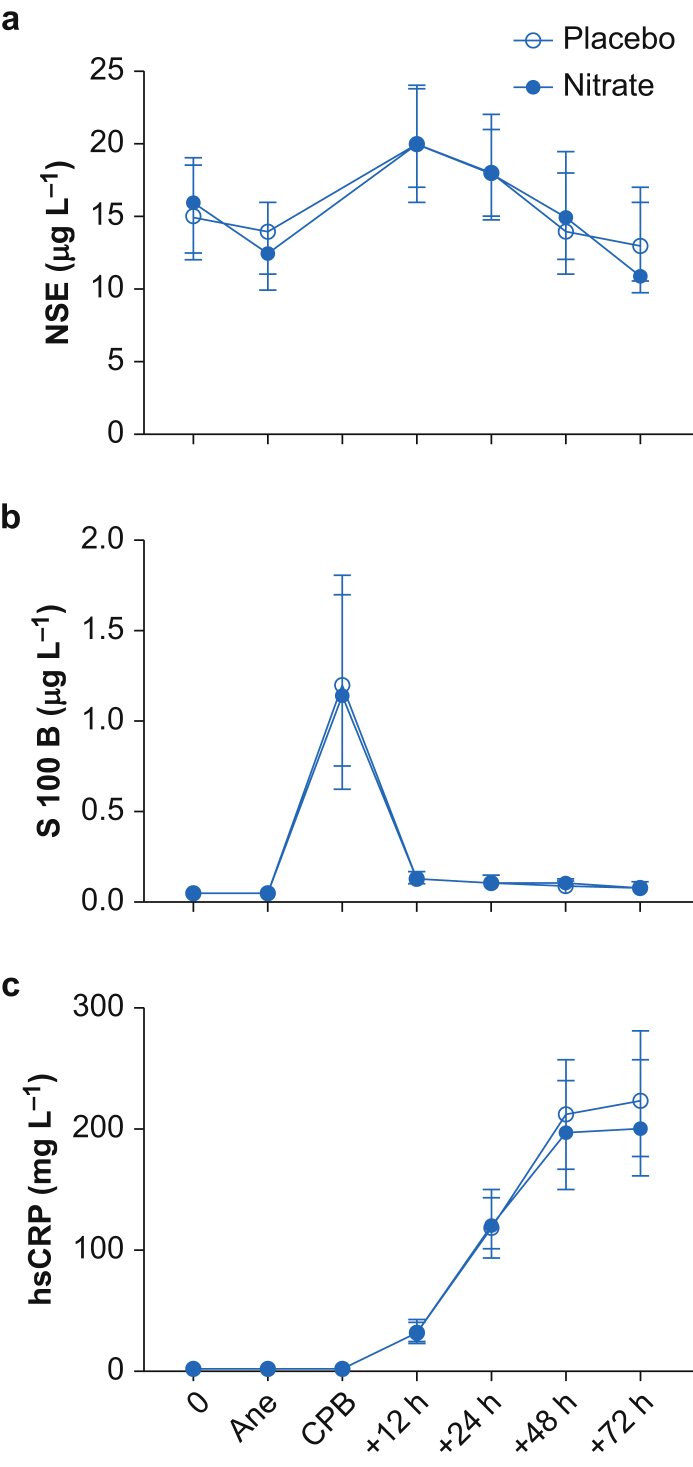
Neuron specific enolase (NSE), S100B protein (S100B), and high sensitivity C-reactive protein (hsCRP) during the 72-h perioperative period. 0=baseline. Data are shown in median and inter-quartile range (IQR). Ane, start of anaesthesia; CPB, end of cardiopulmonary bypass.

**Supplement Figure 1:**
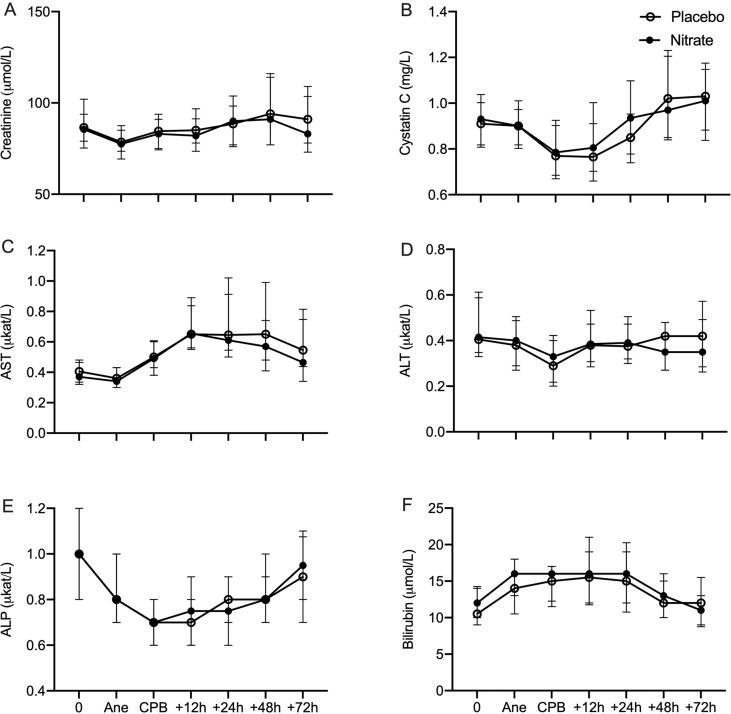
Renal and hepatic biomarkers, Creatinine, Cystatin C, alanine aminotransferase (ALT), aspartate aminotransferase (AST), alkaline phosphatase (ALP) and bilirubin during the 72-hour perioperative period. 0 = baseline, Ane = start of anaesthesia, CPB = end of cardiopulmonary bypass. Data are shown in median and interquartile range (IQR).

Renal and hepatic biomarkers were measured before and after the cardiothoracic surgical event with no differences between nitrate-treated subjects and the placebo group (Supplementary Fig. S1). In addition, the cerebral injury biomarkers NSE and S100B had a similar progress in both groups (Fig. 4).

The inflammatory marker hsCRP increased in both groups with a peak 72 h after extracorporeal circulation (ECC) (Fig. 4). The values at 48 and 72 h were slightly trended to be lower in the nitrate-treated patients, but the difference was not significant in the mixed model analysis (*P*=0.08).

### Perioperative cardiovascular parameters and blood loss

**Supplement Figure 2:**
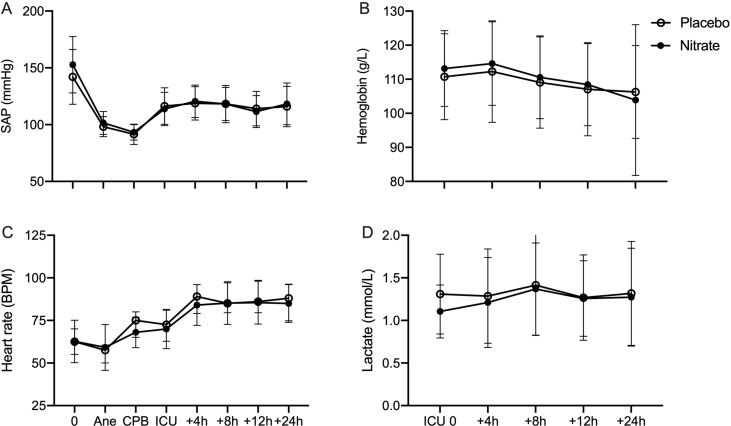
Circulatory parameters and laboratory values during the perioperative period of 24 hours. SAP= systolic artery blood pressure. 0 = baseline, Ane = start of anaesthesia, CPB = end of cardiopulmonary bypass, ICU = Intensive care unit. Data are shown in mean and standard deviation (SD).

The perioperative process with anaesthesia, extracorporeal circulation, and postoperative intensive care affected the hemodynamic parameters as expected, with no effect by the intervention (Supplementary Fig. S2). Postoperative concentrations of haemoglobin and blood lactate were the same in both patient groups. As a result of postoperative complications, some patients needed a prolonged observation period in the ICU, with no difference between the groups (Supplementary Table S1).

**Fig 5:**
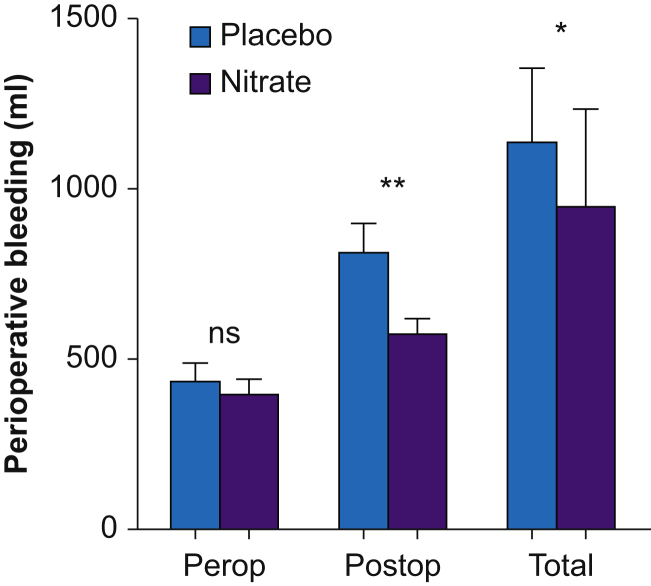
Perioperative (Perop), postoperative (Postop), and total summarised (Total) blood loss in subjects receiving placebo or nitrate, shown in median and inter-quartile range. ∗*P*<0.05, ∗∗*P*<0.01.

Total perioperative blood loss was lower in the nitrate-treated subjects compared with placebo (*P*=0.03, Fig. 5). This difference was mainly attributed to a lesser blood loss in the nitrate-treated subjects in the postoperative period. The number of transfusions of packed red blood cells (RBCs) did not differ between the groups, but the patients in the placebo group required higher amounts of platelets (placebo; 79 ml [199], nitrate; 8 ml [47], *P*=0.03) and plasma transfusions (placebo; 66 ml [207], nitrate; 0 ml, *P*=0.025). Reoperation because of bleeding was performed in two patients in the placebo group and in none in the nitrate group.

## Discussion

In this study we investigated the effect of preoperative nitrate administration on biomarkers of organ injury in patients exposed to on-pump CABG. Despite a marked increase in plasma nitrate and nitrite in the treatment group, we did not observe any protective effects on troponin T release or other injury biomarkers of the heart, kidney, liver, or brain. Although not a prespecified outcome parameter, perioperative bleeding was significantly reduced in the nitrate-treated subjects.

The reason for this study was previous results in animal models of IR injury^15^^,^^16^^,^^32^ and in patients with acute myocardial infarction^28^ showing promising protective effects of inorganic nitrate or nitrite. The rationale for choosing CABG surgery for a ‘proof-of-concept’ study on human IR injury is the scheduled nature of the procedure, allowing preoperative administration of nitrate. We chose plasma troponin T as the primary outcome measure since it is an established marker of myocardial injury and has been associated with morbidity and mortality after CABG.^4^^,^^5^

Several human studies have shown beneficial cardiovascular and metabolic effects of dietary nitrate including lowering of BP,^23^^,^^33^ improved endothelial function,^25^^,^^34^ reduced resting metabolic rate,^35^ and enhanced aerobic efficiency during exercise.^36^ In animal studies, nitrate and nitrite protect against IR injury in the heart,^20^ liver,^16^ kidney,^37^ and brain.^38^ A few studies in humans have shown protective effects of nitrate supplementation on vascular IR injury,^23^^,^^39^ but the present study is the first testing nitrate on cardiac IR injury in humans.

There may be several reasons why we did not see any beneficial effects in the present study. Even though the dose used here and the increase in plasma nitrate and nitrite is comparable to what has been shown to induce cardiovascular and metabolic effects, it might have been too low to be effective in this specific situation. Some studies indicate that more prolonged administration might increase the efficacy of dietary nitrate supplementation.^40^ Another possibility might be that these anions are scavenged during CPB, but this seems unlikely since plasma concentrations at the end of extracorporeal circulation were only slightly reduced, in accordance with known half-lives of these anions.^13^ An interesting observation is that even though the patients were intubated and under anaesthesia, which theoretically would impair oral conversion of nitrate to nitrite and swallowing of nitrite-containing saliva,^42^ plasma nitrite was still elevated several hours into surgery. This may implicate that nitrate can be converted to nitrite somewhere else in the body outside the oral cavity. Indeed, nitrate reduction by a mammalian nitrate reductase has been suggested in the liver^43^ and by bacteria in the small intestine.^44^

Volatile anaesthetics, opioids, adrenergic drugs, and insulin, which were used during surgery in this study, have been afforded cardioprotective effects during cardiac surgery via several mechanisms including the potassium ATP (K_ATP_) channel, mitochondrial permeability transition pore (mPTP), ROS production, and through cytoprotective Akt and extracellular signal kinases (ERK) pathways.^45^ Some of these mechanisms have also been attributed to nitrate and nitrite, which could explain the lack of effects on cardiac IR injury in this study.

Another interesting aspect on the lack of effects on myocardial IR injury in the present study could be the erythrocyte. Recent studies have shown that erythrocytes, during hypoxic conditions, can exert protective effects in models of myocardial IR injury, through the release of NO bioactivity.^46^ A possible mechanism for such NO bioactivity export is through deoxygenated haemoglobin, which can function as a nitrite reductase, yielding NO and other reactive nitrogen species.^47^ In our patients, the heart was perfused with oxygenated blood mixed with cardioplegic solution. Any red blood cell (RBC)-dependent nitrite reduction to NO which is normally potentiated during hypoxia or hypoxia-induced release of a protective agent from the RBC could have been greatly impaired in the oxygenated blood delivered during cardioplegia. Speaking against this explanation is the fact that nitrite has been shown to have preconditioning effects in experimental models^48^ and has also been suggested to convey the effects of remote preconditioning.^19^ In addition, we did not see any effects in the kidney and liver biomarkers, although the IR injury seemed not very profound in these organs.

Another reason for the lack of protection might be that most patients in the study were on multiple medications because of cardiovascular and metabolic disease. Among these drugs, angiotensin converting enzyme (ACE) inhibitors and angiotensin receptor blockers are of special interest since they reduce the action of angiotensin II (ANG II). Among its actions, this hormone stimulates NOX activity and production of ROS. Interestingly, several studies have shown that inorganic nitrate can inhibit NOX activity,^49^ downregulate AT receptors and reduce superoxide generation during IR injury.^50^ Since nitrate and inhibitors of ANG II signalling share a similar mechanism, it may be that no additional effects could be afforded by nitrate. A similar reasoning could be argued with metformin, which shares similar mechanisms as nitrate for its metabolic effects, thereby attenuating any effect of nitrate.^26^

Blood loss was not a predefined outcome parameter, but we found that perioperative bleeding was significantly lower in the patients receiving nitrate and these patients received less plasma and platelet transfusions compared with placebo. Platelets were administered by the attending and blinded anaesthesiologist upon clinical signs of bleeding and not by platelet count. We have no clear explanation for these findings, but we speculate that nitrate could have a protective effect against platelet activation and deposition in the extracorporeal circuits. Loss of platelets and impaired platelet function are thought to contribute to haemostatic defects upon CPB.^51^^,^^52^ Nitrite and NO inhibit platelet aggregation to various stimuli and may therefore inhibit activation, aggregation, and deposition of platelets in the extracorporeal circuits during CPB. Early studies using NO gas to ameliorate platelet activation during CPB are conflicting,^53^, ^54^, ^55^ and the use of NO gas or NO donors has not been adopted in clinical routine. It is noteworthy that nitrate gives rise not only to NO, but also other reactive nitrogen species, making the comparison to previous studies using NO gas not fully valid. We did not follow up on post-surgical platelet count in a standardised manner and further studies are needed to test the hypothesis of a putative platelet protective effect by nitrate during CPB. A difference in postoperative bleeding may also depend on differences in cardiovascular parameters such as BP. However, we did not find any difference in these parameters that could explain our finding.

This study has some limitations. The limited number of patients does not allow for any subgroup analysis regarding sex, dose in relation to weight, and more. As discussed earlier, only two doses of nitrate were used (night before and morning before surgery) and the questions still stands whether a higher dose or a prolonged pretreatment period would have yielded another outcome. Moreover, we did not follow the patients longer term to investigate any effects on parameters such as myocardial salvage index or major adverse cardiovascular events. However, since we did not find any difference between nitrate and placebo in the injury biomarkers and in early adverse events, we believe it is unlikely to find any effects on more long-term parameters.

To conclude, despite numerous preclinical studies indicating protection in IR injury by nitrate or nitrite, pretreatment with dietary nitrate to patients undergoing CABG with CPB does not attenuate the increase in cardiac biomarkers of IR injury. The observation of a reduction in perioperative blood loss is of interest, but needs confirmation in further studies.

## Authors' contributions

Data collection: KEE, FE

Data analysis: KEE, FE, EW

Writing: KEE, EW

Revising: all authors

Study design: FE, JL, AFC, JOL, EW

Patient recruitment: FE
