# Supplementary material for: Effects of inorganic nitrate on ischaemia-reperfusion injury after coronary artery bypass surgery: a randomised controlled trial
Source: Br J Anaesth. 2021 Aug 14;127(4):547–55. doi: 10.1016/j.bja.2021.06.046 (PMC8524391; doi:10.1016/j.bja.2021.06.046)
Supplement: Multimedia component 1 [file mmc1.docx]

Supplement Table 1. Cases of adverse events (percentage in brackets) within 30 days

after cardiac surgery.

The groups are compared with Fischer’s exact test.

| Cases of adverse events: | Placebo (*n*=42) | Nitrate (*n*=40) | *P* |
| --- | --- | --- | --- |
| Respiratory complications (%)  Pleural effusion (%)  Respiratory failure (%)  Pneumothorax (%) | 7 (16.7)  5 (11.9)  2 (4.8)  0 | 6 (15)  4 (10)  1 (2.5)  1 (2.5) | 1 |
| Cardiovascular complications (%)  Atrial fibrillation (%)  Myocardial infarction (%)  Graft failure (%) | 4 (9.5)  4 (9.5)  0  0 | 4 (10)  2 (5)  1 (2.5)  1 (2.5) | 1 |
| Infectious complications (%)  Mediastinal infection (%)  Superficial wound infection (%)  Pneumonia (%)  Unclear infection (%) | 4 (9.5)  2 (4.8)  1 (2.3)  1 (2.3)  0 | 9 (22.5)  2 (5)  5 (12.5)  1 (2.5)  1 (2.5) | 0.14 |
| Re-operation (%)  Cardiac tamponade (%)  Bleeding (%) | 2 (4.8)  1 (2.3)  1 (2.3) | 0  0  0 | 0.49 |
| Gastrointestinal complications (%)  Melena (%) | 0  0 | 1 (2.5)  1 2.5) | 0.49 |
| Neurological complications (%)  Delirium (%) | 1 (2.3)  1 (2.3) | 0  0 | 1 |
| Renal complications (%)  Acute kidney injury (%) | 1 (2.3)  1 (2.3) | 0  0 | 1 |
